# Making Hardware Removal Unnecessary by Using Resorbable Implants for Osteosynthesis in Children

**DOI:** 10.3390/children9040471

**Published:** 2022-03-29

**Authors:** Pascal Heye, Christoph Matissek, Clemens Seidl, Marcell Varga, Tamas Kassai, Gergö Jozsa, Thomas Krebs

**Affiliations:** 1 Department of Pediatric Surgery, Pediatric Trauma Surgery, Children’s Hospital of Eastern Switzerland, 9000 St. Gallen, Switzerland; christoph.matissek@kispisg.ch; 2 Department of Trauma and Orthopedic Surgery, Landeskrankenhaus Baden-Mödling, 2340 Mödling, Austria; clemens.seidl@gmx.at; 3 Dr. Manninger Jenő Trauma Center, Department of Pediatric Trauma Surgery, 1081 Budapest, Hungary; drvmarcell@gmail.com (M.V.); kassai.tamas@obsi.hu (T.K.); 4 Department of Pediatrics, Division of Pediatric Surgery, Traumatology and Otolaryngology, University of Pécs Medical Center, 7624 Pécs, Hungary; dr.jozsa.gergo@gmail.com; 5 Department of Pediatric Surgery, Children’s Hospital of Eastern Switzerland, 9000 St. Gallen, Switzerland; thomas.krebs@kispisg.ch

**Keywords:** resorbable, biodegradable, osteosynthesis, implants, children

## Abstract

Introduction: Following osteosynthesis, children generally require a second surgery to remove the hardware. This becomes unnecessary, by using resorbable implants. Limiting the number of required surgeries and their associated risks, this technique provides critical aspects of minimally invasive surgery. This review focuses on resorbable implants for osteosynthesis for the treatment of fractures in children and discusses their clinical features. Method: We provide an overview of the two most common technologies used in resorbable osteosynthesis materials: polymer- and magnesium-based alloys. Clinical examples of osteosynthesis are presented using polymer-based ActivaTM products and magnesium-based Magnezix^®^ products. Results: Polymer-based implants demonstrate surgical safety and efficacy. Due to their elasticity, initial placement of polymer-based products may demonstrate technical challenges. However, stability is maintained over the course of healing. While maintaining good biocompatibility, the rate of polymer-resorption may be controlled by varying the composition of polyesters and copolymers. Similarly, magnesium-based implants demonstrate good mechanical stability and resorption rates, while these characteristics may be controlled by varying alloy components. One of the significant shortcomings of magnesium is that metabolism results in the production of hydrogen gas. Both technologies provide equally good results clinically and radiographically, when compared to non-resorbable implants. Conclusion: Resorbable osteosynthesis materials demonstrate similar therapeutic results as conventional materials for osteosynthesis. Resorbable implants may have the potential to improve patient outcomes, by sparing children a second surgery for hardware removal.

## 1. Introduction

By what parameters do we measure how minimally invasive *Minimally Invasive Surgery* really is? By the size of the incision, by the time in the operating room, by the risks of collateral damage, by the burden to the patient, or by the necessity of multiple surgeries?

Ideally, *Minimally Invasive Surgery* aims to reduce all the above-mentioned issues. Using thoracoscopy, laparoscopy or arthroscopy surgeons strive to make surgery as beneficial as possible for patients. Applying the same parameters mentioned above to surgical techniques not using video-assisted techniques, should they not also qualify as *Minimally Invasive Surgery*?

By using resorbable implants, a second surgery to remove the hardware becomes unnecessary. Sparing children from a second anesthesia, another incision, and a new course of healing, resorbable implants may have the potential to improve patient outcomes, by providing one single definitive surgery.

This article focuses on resorbable implants for osteosynthesis for treatment of fractures in children and discusses their clinical advantages and disadvantages.

## 2. Background

While most fractures in children can be treated nonoperatively, selected cases of unstable, comminuted, and open fractures require surgical treatment. Surgery involves closed or open reduction of the fracture, followed by osteosynthesis. To date, osteosynthesis materials made from non-resorbable metal alloys demonstrate excellent biocompatibility and great stability and are considered the gold-standard. Osteosynthesis materials in pediatric surgery most commonly consist of K-wires, screws, plates, and intramedullary nails made from stainless steel or titanium. Surgical stainless steel consists of iron, chrome, and nickel, while titanium alloys are made of aluminum, vanadium, and niobium. While microparticles found in the surgical field were believed to be harmless, some data suggest a potential to trigger immune reactions and allergies [1,2,3].

Following the healing of the fracture, hardware removal is generally recommended in the pediatric population, due to concerns of interference with growing bones, and potential risks of infection, soft-tissue irritation, and nerve-injury [4]. This second surgery is generally recommended one to twelve months after osteosynthesis (depending on the materials used, the fracture pattern, and the age of the patient), when successful consolidation of the fracture was documented. Although the performance and biocompatibility of metal implants are satisfactory, there are associated disadvantages and risks associated with hardware removal. These may include surgical risks, such as infections or soft tissue injuries [5,6], and risk attributed to anesthesia. Especially in the pediatric population, the risk of growth arrest is also increased by the removal of implants placed near growth plates. Therefore, these surgeries should not be underestimated. Alongside the disadvantages for patients, hardware removal generates a significant cost for healthcare systems. Multiple surgeries, more frequent clinic visits, and increased staff demands are a strain to already busy providers.

In light of these reasons, a strong interest is demonstrated to develop biodegradable implants, making further surgery for hardware removal unnecessary.

### 2.1. Materials Used

Demands for any osteosynthesis materials are that surgery should be minimally invasive, the fixation should provide lasting stability to the reduction, and there is minimal associated risk for complications. The critical shortcomings of biocompatibility and stability remain the two most crucial aspects of resorbable osteosynthesis materials. First reports on resorbable osteosynthesis materials (rOSM) in the pediatric trauma population were published in the nineties [7,8]. Since then, major developments to improve these characteristics resulted in a range of technologies studied and applied. Two technologies used in rOSM are polymer- and magnesium-based alloys.

### 2.2. Polymer-Based Implants

Bioresorbable polymers consist of polyester with repeating units of α-hydroxy acid derivates. Degradation of the polymers takes place in the citric acid cycle, where hydrolysis breaks the ester bonds to form water and carbon dioxide [9]. This reaction will cause the local pH to drop, creating an acidic environment around the implant.

Currently, the most commonly used polymer is poly-lactic acid (PLA) and its derivate PLGA (poly lactide-co-glycolide). PLGA belongs to the second generation of bioresorbable materials, being constructed as a copolymer of L-lactide and glycolide. While this material exhibits increased stability it also has shorter absorption times. These times may be varied, depending on the composition of the copolymer, thereby achieving a slow enough decomposition time to achieve high biocompatibility [10]. For PLGA, resorption times of 9 to 12 months were reported [11].

In their beginning, polymer-based implants were of particular interest in maxillofacial surgery, and have since undergone great advances to accommodate for use in areas of strong forces, such as the mandible [12]. Of particular note was a self-reinforcing technology, that increased strength and stiffness, thereby extending the spectrum of clinical applications [13].

In this review, reference is made to PLGA-based products. Intramedullary Activa IM-Nail^TM^ (Bioretec Ltd., Tampere, Finland) is available in 2.0, 2.7, and 3.2 mm diameters, and is 200, 300, or 400 mm long, with X-ray sensitive markers on both ends. ActivaPin^TM^ is available in 1.5, 2.0, 2.7, and 3.2 mm diameters, and is 50 or 70 mm long. ActivaScrew™ is available fully threaded in 2.0, 2.7, 3.5, and 4.5 mm diameter, and 20 to 90 mm long. Cannulated screws are also available.

### 2.3. Magnesium-Based Implants

First investigations on magnesium-based implants were reported by Lambotte in 1932 [14]. Ever since, significant development has occurred, and today magnesium alloys such as MgYREZr (magnesium, yttrium, rare earth elements, zirconium) are used and studied. This specific alloy consists mainly of WE43, an aluminum free component, that when combined with rare earth elements, results in a high-strength magnesium alloy. Other magnesium alloys free of rare earth elements are currently undergoing in vitro and in vivo studies, with equally positive results [15,16].

During the metabolism of magnesium alloys, hydrogen gas is produced [17]. This is a result of the corrosion reaction, in which Mg + 2H_2_O → Mg(OH)_2_ + H_2_. The gas diffuses into the local tissues and blood. Once these tissues are saturated, gas accumulates in tissue cavities, such as cancellous bone [18] (Figures 3 and 5). Alloying components may contribute to a galvanic corrosion, resulting in a more rapid corrosion process, thereby forming increased amounts of hydrogen gas, as well as changes in local pH [19]. These are among the most critical aspects for successful application of magnesium-based materials. By mixing alloys and coatings, developers focus on controlling the rate of degradation, as a fast corrosion may lead to increased gas formation and inhibition of osteoblasts, risking implant failure and injury to the physis [20]. Meanwhile, several animal studies demonstrated that there were no side-effects due to metabolites, and that the degradation process was slow and homogenous [21,22]. Due to their mechanical stability and slow resorption, magnesium-based implants are designed for use in intra- and extraarticular fractures. Clinical reports demonstrate satisfactory surgical results and clinical outcomes in orthopedic as well as trauma surgery [7,23,24,25,26].

In this review, reference is made to magnesium-based Magnezix^®^ CBS screws (Syntellix AG, Hannover, Germany). The product is available in 2.0, 2.7, and 3.5 mm diameters, and 6 to 40 mm long. A cannulated Herbert screw (Magnezix^®^ CS) is available in 2.0 to 4.8 mm diameters, and 8 to 70 mm long. Magnezix^®^ Pins are available in 1.5 to 3.2 mm diameters, and 8 to 50 mm long.

### 2.4. Clinical Applications

As of the publication of this manuscript, surgical safety and therapeutic efficacy of the following products are studied in ongoing prospective clinical trials (data not published). All studies were approved by the local ethics committee.

#### 2.4.1. Fracture of the Tibial Tuberosity

**Figure 1:**
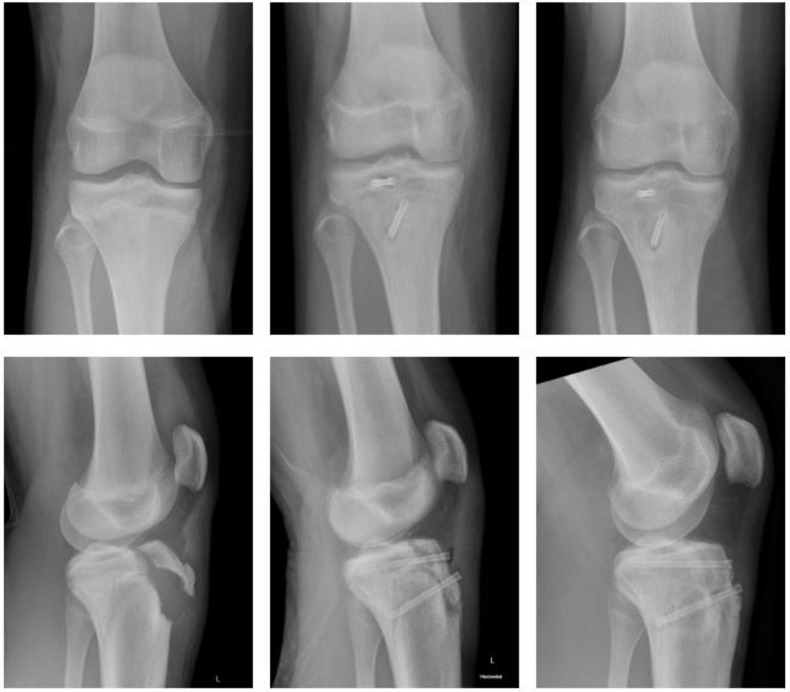
Fracture of tibial tuberosity with Magnezix^®^ screw: preop, 2 months postop, 10 months postop (left to right).

Magnesium (Figure 1).

A fourteen-year-old male sustained a fracture of the tibial tuberosity (Ogden Type III B). He underwent open reduction and internal fixation (ORIF) the following day. Magnezix^®^ CS screws were used: 4.8 × 50 mm in the metaphysis, 4.8 × 55 mm in the epiphysis. The postoperative course was uneventful with unremarkable wound-healing. The knee was immobilized for 4 weeks using a brace, with a subsequent gradual increase in range of motion (ROM) until 12 weeks after surgery. There was no clinical impairment.

Polymer (Figure 2).

In a similar case, a fourteen-year-old male sustained a tibial tuberosity fracture (Ogden Type III B). He also underwent ORIF. The fracture was stabilized using three resorbable screws (ActivaScrew™, 4.5 × 40 mm) and a resorbable polydioxanone suture. The postoperative course was uneventful. The knee was immobilized in a long rigid brace, with full weight bearing postop. ROM was gradually increased for six weeks in the brace. At 12 weeks postop the patient demonstrated no clinical impairment.

#### 2.4.2. Fracture of the Medial Epicondyle

**Figure 3:**
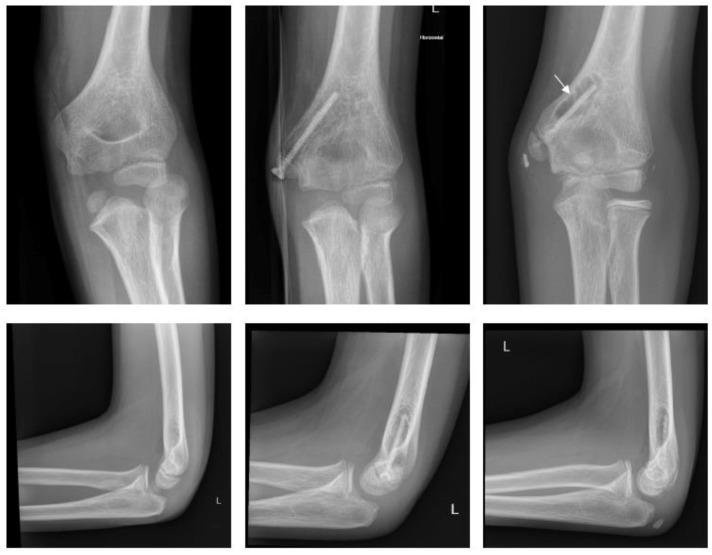
Fracture of medial epicondyle with Magnezix^®^ screw: preop, 1 month postop, 12 months postop (left to right). Arrow marking gas formation.

Magnesium (Figure 3).

A ten-year-old boy with dislocation of the elbow sustained a medial epicondylar fracture. The epicondyle was trapped in the joint. The patient underwent ORIF using a Syntellix Magnezix^®^ CBS screw (3.5 × 38 mm). The postoperative course was uneventful. At 3 months postop, the patient demonstrated a 10° flexion deficit, which had completely resolved one year after surgery.

Polymer (Figure 4).

A six-year-old girl with dislocation of the elbow sustained a fracture of the medial epicondyle. After reduction of the elbow, she underwent ORIF the same day. Two bioresorbable PLGA pins (ActivaPin^TM^ 2 mm) and a looped polydioxanone suture were used. The postoperative course was uneventful. The elbow was immobilized with restricted ROM in a hinged brace for 3 weeks. Subsequently the patient demonstrated no clinical impairment with full ROM 3 months postop.

#### 2.4.3. Fracture of the Lateral Condyle

**Figure 5:**
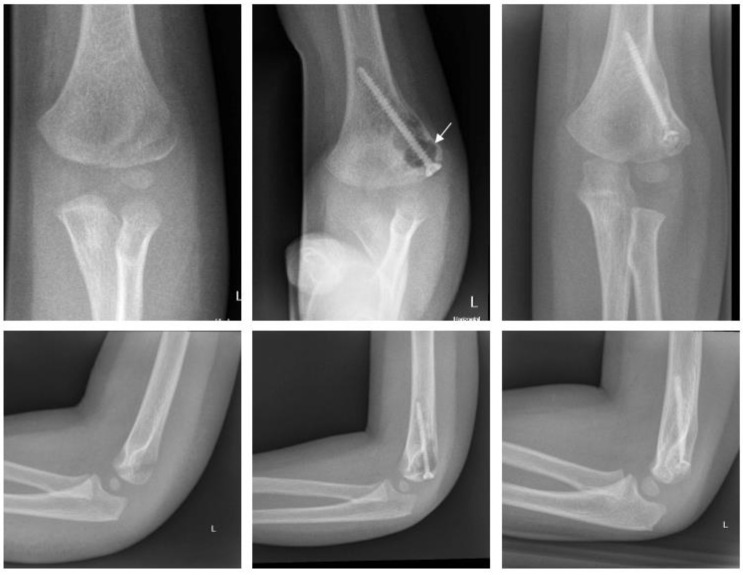
Fracture of lateral condyle with Magnezix^®^ screw: preop, 1 month postop, 12 months postop (left to right). Arrow marking gas formation.

Magnesium (Figure 5).

A two-year-old boy sustained a displaced fracture of the lateral condyle. ORIF was performed using a Syntellix Magnezix^®^ CBS screw (3.5 × 40 mm). The postoperative course was uneventful. One year after surgery the patient demonstrated no complaints, with full ROM and physiologic axis of the arm.

Polymer (Figure 6).

An eight-year-old boy sustained a displaced lateral condyle fracture. He underwent ORIF the same day, using two resorbable PLGA pins (ActivaPin^TM^ 2 mm) and a looped polydioxanone suture. The postoperative course was uneventful. The elbow was immobilized for 3 weeks in a brace. There was no clinical impairment.

#### 2.4.4. Fracture of the Ankle

Polymer (Figure 7).

A thirteen-year-old girl sustained a displaced triplane fracture. She underwent ORIF the same day. Two bioresorbable PLGA screws (ActivaScrew^TM^ 3.5 mm) were used. The postoperative course was uneventful. The lower limb was immobilized in a short cast for 6 weeks with gradual increase of weight bearing until 12 weeks after surgery. There was no clinical impairment.

#### 2.4.5. Fracture of the Forearm Shaft

Polymer (Figure 8).

An eight-year-old girl sustained a displaced forearm shaft fracture. She underwent closed reduction and internal fixation the same day. Two bioresorbable PLGA-based intramedullary nails (Activa IM-Nail^TM^ 3.2 and 2.7 mm) were used. The postoperative course was uneventful. The upper limb was immobilized in a long cast for 3 weeks, followed by a short cast for two weeks. There was no clinical impairment.

## 3. Discussion

### 3.1. Biocompatibility

Biodegradability is regarded as a major concern in resorbable surgical materials. Various complications such as fistula formation, osteolysis, soft tissue inflammation and foreign body reaction have been reported [27]. Significant improvement was achieved by developing PLGA polymers, subsequently to PLA. Polymers with a decreased amount of low-molecular weight components demonstrate a lower rate of lactic acid release, resulting in a less significant drop in local pH, thus preventing extensive tissue inflammation and toxic responses [28,29]. A multitude of studies has proven surgical safety and efficacy in the use of PLGA in adult maxillofacial surgery [12]. Yerit et al. reported on copolymer PLDLA-based implants used in 13 children with mandibular fractures, with excellent fracture healing and no adverse reactions during a 26 months follow-up period [30]. Similarly, the use of PLGA in pediatric mandibular fractures was reported as effective as safe [31]. Although multiple studies evaluated the use of different polymer-based implants, to date, no randomized controlled study analyzed which polymer is better in the pediatric population.

The metabolism of magnesium-based alloys is slow, thereby providing sustained stability during fracture healing and remodeling. One of the significant shortcomings of magnesium, however, is that the metabolism results in the production of hydrogen gas [32]. This may lead to cavity formation around the implant with a potential to cause hardware loosening. This risk may be amplified by osteoclast-induced bone resorption. Gas formation was associated with local soft tissue gangrene and wound disturbances in selected cases and animal models [33]. However, although lucency on radiographs suggests significant gas formation, histopathology of these uncalcified sections provides proof of accumulating calcium phosphate, and enhanced mineral apposition and periosteal bone growth, implying an osteoblastic response to magnesium [34]. In vitro studies showed that magnesium alloys and their metabolites are well tolerated by osteoblasts and growth plate chondrocytes [35]. Biocompatibility was demonstrated to be improved by surface modification [36]. Micro-arc oxidation surface treatment could have the potential to lower corrosion rates, as demonstrated in animals models [37]. Further research on magnesium-coatings is necessary to demonstrate potential long-term effects of corrosion on the growth plate.

### 3.2. Stability

Two factors are critical in evaluating the biomechanical properties of resorbable implants: tensile strength (TS, maximum load a material can support without fracture) and elastic modulus (EM, resistance to being deformed elastically). The combination of a high TS and a moderately low EM, assures that the implant resists forces exerted by weight bearing, while allowing plastic elasticity. Polymer-based PLGA demonstrates a TS of 63 MPa and an EM of 2.7 GPa [38]. Magnesium-based WE-43 has a TS of 295 MPa and an EM of 44 GPa [39]. In comparison, cortical bone has a TS of 35–283 MPa and an EM of 5–23 GPa [40], while stainless steel has a TS of 490 MPa and an EM of 193 GPa [41]. This suggests that polymer-based implants are elastic and should not be used in early weight-bearing rehabilitation, while magnesium-alloys are highly resistant to breaking, and suitable for use in extra- and intra-articular fractures.

Due to lacking mechanical strength and stability in clinical practice, implantation of a polymer-based nail requires a metal nail to be inserted into the medullary canal first, to form the tract for the polymer implant. This metal nail is then replaced by the PLGA nail. Due to their elasticity, PLGA nails cannot maintain a curved form and thereby support a reduction, as in pre-bend titanium elastic nails (TEN). Even though Activa^TM^ products demonstrate a 1% volume increase 24 h after insertion which increases stability, additional casting is required to maintain reduction and alignment. This demonstrates a significant disadvantage compared to TEN, as they, generally, do not require additional cast immobilization.

However, in vivo studies demonstrated satisfactory stability and patient outcomes comparing PLGA-based intramedullary nails with TEN [42]. It was demonstrated that biodegradable intramedullary nails provide similar results regarding long-term ROM. The authors also reported excellent radiographic bone union at two years post-surgery, and no difference between the groups. While hospitalization time was similar, operating time was significantly higher in the rOSM group compared to TEN (mean 80 min vs. mean 53 min, *p* = 0.014). Equally satisfying results were demonstrated using polymer-based implants for the treatment of radial condyle [43], medial epicondyle [44], supracondylar humerus [45], and patellar fractures [46].

Baldini et al. provided a systemic review, highlighting the clinical context of magnesium-based implants [47]. Of the 20 studies included, 19 were based on adult data, while 1 study enrolled patients under the age of 18 years. All comparative studies demonstrated that treatment with magnesium-based implants was not inferior compared to conventional metal implants. All single cohort studies yielded good clinical results with no noteworthy complications. From this review, only one case report reported implant failure requiring revision surgery [48]. Additionally, Baldini et al., reported a case series including 14 pediatric patients undergoing a range of orthopedic as well as fracture osteosynthesis with Magnezix^®^ screws. They reported good clinical and radiographic fracture healing, with no adverse reactions. In another study, May et al., reported on their experience comparing Magnezix^®^ screws to conventional titanium screws in fracture fixation [26]. Radiographic streaks of gas were commonly observed in the surgical field and disappeared within 2 to 3 months. Between the fourth and sixth month, gas formation started to be visualized within the bone along the implant. Two years after surgery, gas was no longer demonstrated on radiographs, CT, or MRI. Fracture union was achieved in all patients without major complications. Similarly, previous case series reported good clinical results and fracture healing using magnesium-based implants in the surgical treatment of fractures [25,49]. However, caution must be practiced when evaluating the potential risks associated with magnesium-based screws. Klauser reported a series of 100 hallux valgus osteotomies with magnesium screws. Three patients demonstrated delayed wound healing, two had deep surgical site infections, and one screw fracture was reported [50].

Although it was demonstrated that magnesium-based implants feature superior biomechanical stability compared with polymer-based implants [51,52], this is true only for selected cases of fracture patterns and locations. Since both technologies have specific advantages when used in different locations (epi-, meta-, or diaphyseal), we do not recommend an observational comparison of these two technologies, given the distinct differences in fracture pathophysiology. However, we encourage that these two technologies should be studied in direct comparison, to evaluate the specific pros and cons in different fracture patterns.

Another critical aspect in evaluating socioeconomic efficacy of rOSM goes beyond clinical observations. rOSM are generally more expensive than conventional implants, due to the materials used and the processes of production and sterilization. However, cost analyses suggest a financial advantage of resorbable materials when calculating total costs of treatment, including frequency of clinic visits, surgery for hardware removal, and costs associated with morbidity. Two studies from Finland demonstrated, that by using resorbable implants in osteosynthesis of ankle fractures, average costs saved per patient were approximately 380 to 1300 US$ [53,54].

## 4. Conclusions

rOSM demonstrate similar therapeutic efficacy and surgical safety as conventional osteosynthesis materials. By successfully implementing resorbable implants, a second surgery to remove the hardware becomes unnecessary. Sparing children from a second anesthesia, another incision, and a new course of healing, resorbable implants may have the potential to improve patient outcomes, making pediatric fracture surgery as minimally invasive as possible.

However, there remain several limitations, such as concerns about mechanical stability and biocompatibility. To date, literature on the use of rOSM in children is limited to small sample size studies. Future prospective, larger scale polymer-based research should aim to improve mechanical strength, while magnesium-based research mandates to control corrosion rates and improve gas formation. It will be critical to evaluate the long-term effects potential metabolites may have on the growing skeleton. Until then, we recommend that the surgeon considers the use of rOSM, while always maintaining the option to convert to conventional implants.

## Figures and Tables

**Figure 2:**
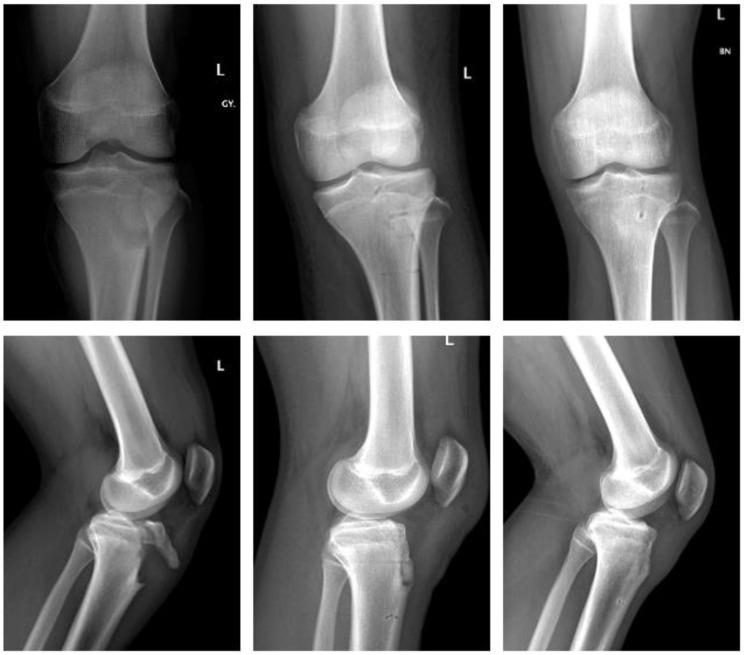
Fracture of tibial tuberosity with ActivaScrew™: preop, 2 days postop, 6 months postop (left to right).

**Figure 4:**
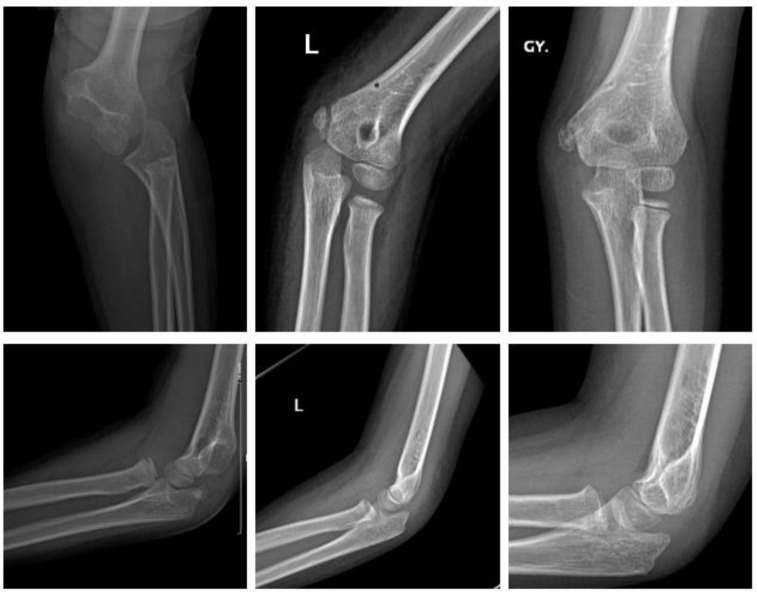
Fracture of medial epicondyle with ActivaPin^TM^: preop, 1 day postop, 6 months postop (left to right).

**Figure 6:**
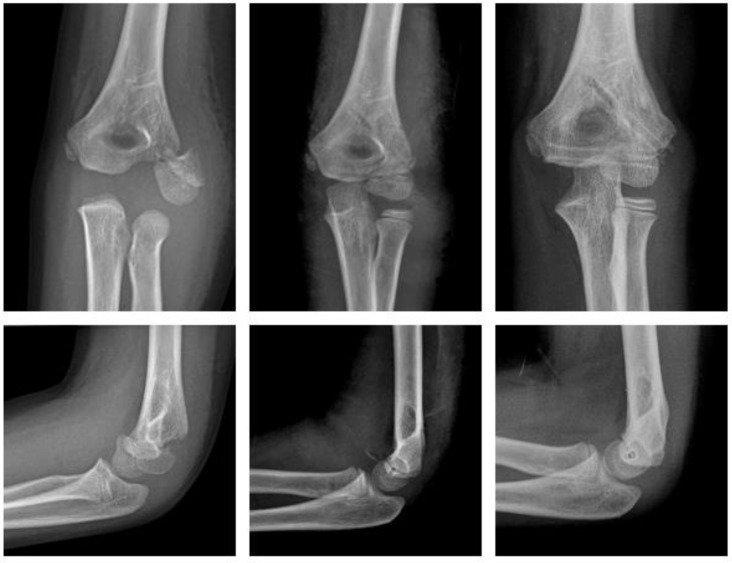
Fracture of the lateral condyle with ActivaPin^TM^: preop, 1 week postop, 6 months postop (left to right).

**Figure 7:**
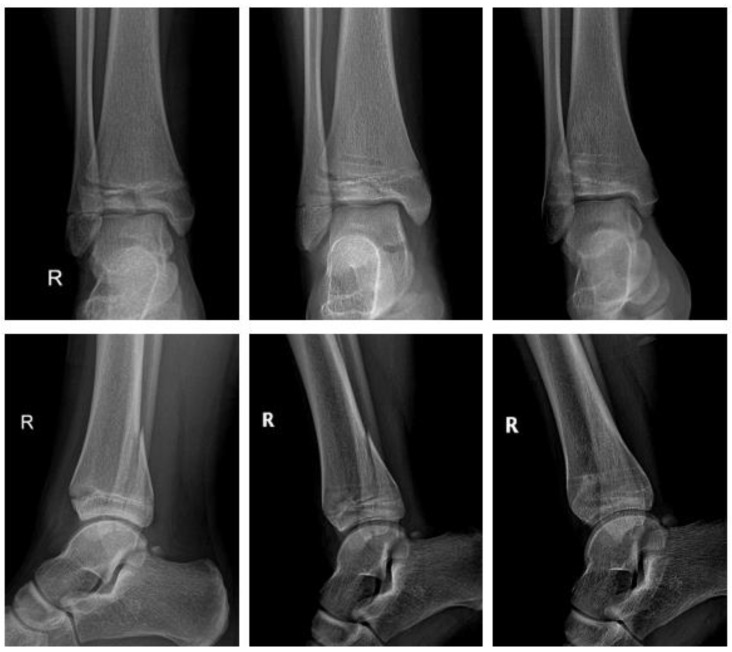
Triplane fracture with ActivaScrew^TM^: preop, 1 week postop, 6 months postop (left to right).

**Figure 8:**
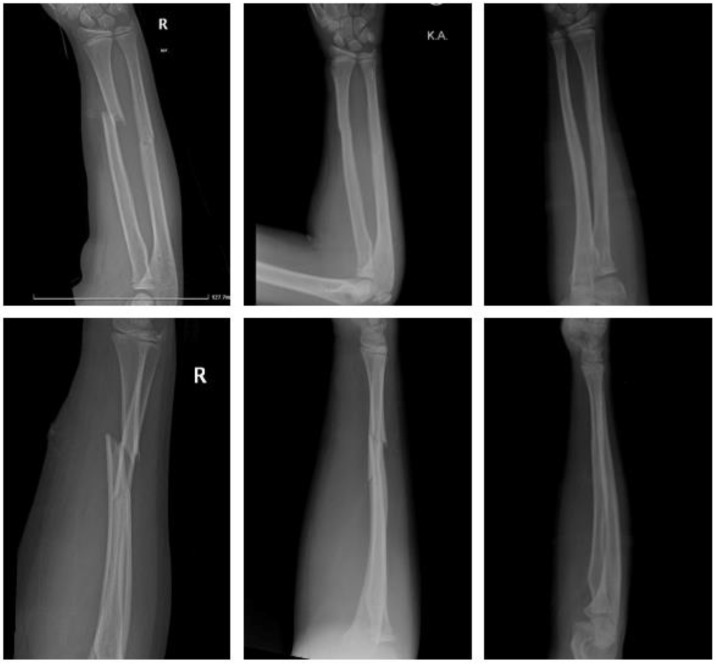
Forearm shaft fracture with Activa IM-Nail^TM^: preop, same day postop, 6 months postop (left to right).
